# Myocardial fibrosis and viability: the role of imaging and biomarkers in patients with chronic total occlusion on coronary angiography

**DOI:** 10.11613/BM.2026.020503

**Published:** 2026-06-15

**Authors:** Željka Dragila Tomašić, Lea Maršić, Anamaria Mikolčić, Lana Maričić, Kristina Selthofer Relatić, Darija Šnajder Mujkić

**Affiliations:** 1 Department of Emergency Medicine, University Hospital Centre Osijek, Osijek, Croatia; 2 Faculty of Medicine, Josip Juraj Strossmayer University of Osijek, Osijek, Croatia; 3 Department of Emergency Medicine, General Hospital Našice, Našice, Croatia; 4 Division of Cardiology, Department of Internal Medicine, University Hospital Centre Osijek, Osijek, Croatia; 5 Clinical Institute for Nuclear Medicine and Radiation Protection, University Hospital Centre Osijek, Osijek, Croatia

**Keywords:** biomarkers, collagen, coronary artery disease, fibrosis, galectin 3

## Abstract

In patients with chronic total occlusion on coronary angiography, myocardial viability assessment is an essential tool in guiding treatment decisions, especially revascularization. Current non-invasive tests are often limited by the presence of myocardial fibrosis. This narrative review incorporates evidence from 76 studies and aims to provide an updated overview of non-invasive methods for myocardial viability assessment, with emphasis on the role of myocardial fibrosis. Every imaging modality provides valuable information, even though they differ in sensitivity, specificity, availability, radiation exposure, contraindications and limitations in the presence of fibrosis. Biomarkers that provide insight into different aspects of myocardial fibrosis – including collagen synthesis (procollagen type I carboxy-terminal propeptide and procollagen type III amino-terminal propeptide), as well as inflammation and fibrotic signalling (galectin-3) – may complement imaging by reflecting myocardial remodelling. However, main limitations to this approach are no standardized reference ranges and cut-off values, analytical variability and insufficient validation for clinical outcomes prediction. Despite their potential to guide personalized revascularization decisions, circulating fibrosis biomarkers currently remain experimental tools. Further large-scale prospective clinical studies that incorporate biomarkers with imaging are needed before their implementation into clinical practice.

## Graphical abstract

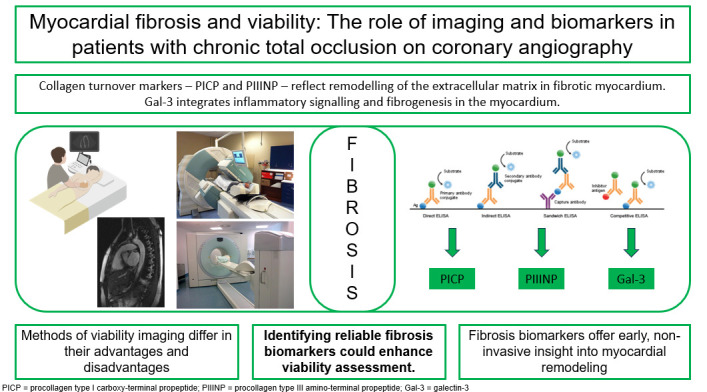

## Highlights

• Viable myocardium guides treatment decisions in chronic coronary disease

• Early detection of myocardial fibrosis may improve patient outcomes

• Terminal collagen propeptides are promising markers of myocardial fibrosis

• Galectin-3 links inflammation and fibrosis in the myocardium

• Identifying reliable fibrosis biomarkers could enhance viability assessment

## Introduction

Chronic total occlusion (CTO) is defined by the CTO Academic Research Consortium as major coronary artery occlusion without forward flow that has been present for ≥ 3 months (*1*). It is found in 15-20% of patients undergoing coronary angiography (*2*, *3*). Although collateral circulation develops to compensate for reduced blood flow, it is often insufficient to fully restore myocardial perfusion (*4*).

The rate and severity of complications for percutaneous coronary intervention in patients with CTO are higher (1-3%) when compared to other procedures in chronic coronary syndrome (*3*, *5*-*7*). However, performing percutaneous coronary intervention in patients with symptoms (angina or dyspnoea) significantly improves their quality of life compared to optimal medical therapy (*8*-*11*).

On the other hand, treatment decisions in asymptomatic patients rely on accurate myocardial viability assessment, which can predict functional recovery after revascularization (*12*, *13*). Non-invasive imaging modalities used for myocardial viability assessment differ in specificity, sensitivity, availability, operator-dependence and radiation exposure (*14*, *15*). Their diagnostic accuracy may also be complicated by the presence of myocardial fibrosis (*16*).

Circulating fibrosis biomarkers – procollagen type I carboxy-terminal propeptide (PICP), procollagen type III amino-terminal propeptide (PIIINP) and galectin-3 (Gal-3) – show promising results in viability assessment by reflecting direct tissue remodelling. They have the potential to identify fibrotic non-viable myocardium earlier and more precisely (*17*, *18*).

The aim of this review is to summarize current non-invasive imaging modalities for myocardial viability assessment, explain the pathophysiological role of myocardial fibrosis and evaluate circulating fibrosis biomarkers as complementary tools for clinical decision-making for revascularization in patients with CTO.

## Methods

This paper is a structured narrative review. A literature search was conducted and finished in May 2025 to identify publications relevant to the role of non-invasive imaging and circulating biomarkers (particularly PICP, PIIINP and Gal-3) in myocardial viability and fibrosis. The search covered publications from January 2005 to May 2025. It was performed using the PubMed/MEDLINE database because it provided the most comprehensive and peer-reviewed indexed sources in imaging and biomarker research relevant to CTO population. Non-indexed databases were excluded to improve clinical relevance and minimize heterogeneity. The following keywords and their combinations using Boolean operators (AND, OR) were used to refine the search: “myocardial viability”, “fibrosis biomarkers”, “myocardial fibrosis”, “imaging”, “chronic coronary syndrome”, “chronic total occlusion”, “PICP”, “PIIINP”, and “Galectin-3”. Titles/abstracts and full texts were screened by two independent reviewers, with disagreements resolved by a senior third reviewer.

Inclusion criteria:

Original research papers, meta-analyses, systematic reviews or guidelines
Studies including adult patients (> 18 years of age) with coronary artery disease, chronic coronary syndrome or heart failure with fibrosis related aetiology
Animal studies that provide insights relevant to fibrosis or myocardial viability
Studies evaluating non-invasive myocardial viability assessment and pathophysiology of myocardial fibrosis that are relevant to circulating fibrosis biomarkers (PICP, PIIINP, Gal-3).

Exclusion criteria:

Non-English articles
Case reports, editorials and conference abstracts
Pediatric or congenital heart disease population
Animal studies without relevance to fibrosis or myocardial viability.

**Figure 1:**
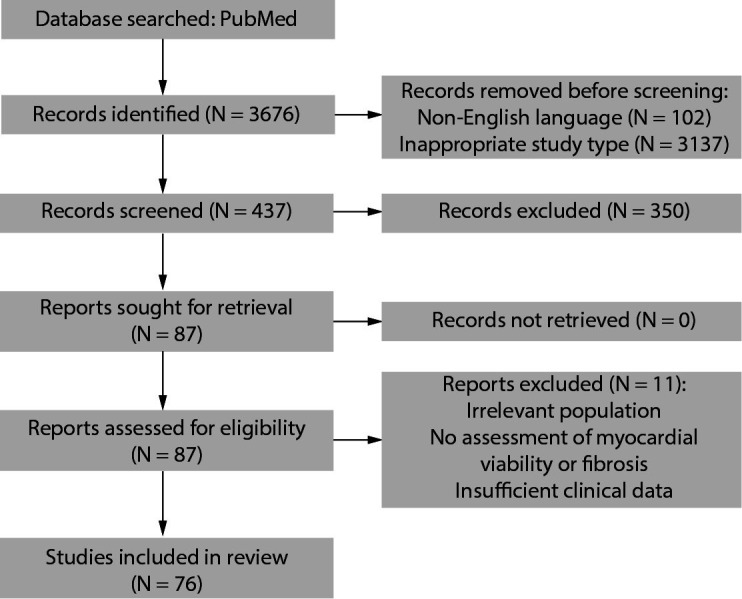
PRISMA-style flow diagram of the literature search and study selection process

After screening for eligibility, 76 studies were included as follows: 13 covering chronic total occlusion, 14 myocardial viability imaging modalities, 11 pathophysiology of myocardial fibrosis, 15 collagen biomarkers and 23 galectin-3. The study selection process is shown in Figure 1.

## Myocardial viability testing

In research and clinical practice, viable myocardium shows contractile cellular dysfunction at rest, which is expected to improve after coronary revascularization. Recurrent ischemia triggers myocardial hibernation – adaptive down-regulation of myocardial function – which exists on a clinical spectrum of reversible ischemic dysfunction (*19*). Myocardial stunning represents impaired contractility following resolution of acute ischemia, lasting for several hours or days, but recovering spontaneously (*20*). Both hibernating and stunned myocardium are considered viable. Viability should be considered as a spectrum, where revascularization of each component may yield diverse pathophysiological benefits (*19*).

The utility of a viability test is judged on its accurate prediction of contractile dysfunction reversal after revascularization using quantitative and/or qualitative measures. Improvement may occur within hours to days in areas of stunned myocardium, or months in areas of hibernating myocardium. Every imaging method focuses on a different element of pathophysiology (*21*). Viability imaging modalities differ in their advantages and limitations (Table 1), but they all play an essential role in the management of patients with chronic coronary syndrome and CTO. The choice should be individualized based on patient characteristics, possible contraindications, expertise and local availability (*22*).

**Table 1:** Comparison of non-invasive imaging modalities for viable myocardium detection

**Test**	**Advantages**	**Disadvantages**
SPECT	Inexpensive Widely available	Exposure to radiation False negatives in the setting of multi-vessel disease False positives due to attenuation
PET	Greater accuracy than SPECT Anatomical information	Exposure to radiation Limited availability and facility expertise
DSE	No exposure to radiation or contrast Structural information Higher specificity in obese and female patients	Dependant on technical expertise and imaging quality Lower sensitivity in the setting of single-vessel disease
CMR	Higher sensitivity and specificity Gives detailed structural information	Expensive, limited availability Contraindicated in advanced renal disease Affected by presence of arrythmia
*Adapted from (20). SPECT - single photon emission computed tomography. PET - positron emission tomography. DSE - dobutamine stress echocardiography. CMR - cardiac magnetic resonance.		

Positron emission tomography (PET) assesses glucose metabolism using glucose analogues, where preserved glucose uptake indicates viable myocardium even when contractility is impaired. It demonstrates the highest sensitivity for detecting preserved metabolic activity and predicting contractile recovery. However, its accuracy depends on standardized preparations (fasting, glycemic control and insulin protocols) and does not directly quantify the scar tissue, which may lead to false positive results in acutely stunned or inflamed myocardium (*23*-*26*).

Single photon emission computed tomography (SPECT) evaluates regional blood flow by quantifying intracellular uptake of perfusion tracers (*e.g*., 99mTc-sestamibi). It is widely used due to its accessibility and familiarity, even though its lower spatial resolution and reduced accuracy in comparison to PET limits its use for detailed viability characterization (*27*, *28*).

Dobutamine stress echocardiography (DSE) assesses contractile reserve by stimulating β1-adrenergic receptors with dobutamine, causing systolic thickening in viable myocardium. It has high specificity for predicting short and mid-term functional improvement after revascularization. However, image quality and interpretability depend on the acoustic window and operator expertise, which may reduce diagnostic accuracy (*15*).

Cardiac magnetic resonance (CMR) evaluates myocardial tissue using late gadolinium enhancement for scar quantification and quantitative mapping (native T1 and extracellular volume) for measurement of diffuse interstitial fibrosis. It provides the most comprehensive structural assessment, but its use may be limited due to availability, as well as in patients with renal dysfunction and implanted devices (*29*, *30*).

A perfect method for myocardial viability testing has not been found. Fibrotic tissue, which results from chronic damage and remodelling, may not accurately reflect the true viability of the heart muscle. This can lead to challenges in distinguishing viable and non-viable tissue, influencing treatment plans. Therefore, it is important to understand the pathophysiology of myocardial fibrosis.

## Myocardial fibrosis

Myocardial fibrosis represents a significant global health problem implicated in nearly all forms of heart disease leading to increased left ventricular stiffness and impaired contraction and relaxation (*31*, *32*). It develops when persistent injury, such as chronic ischemia, pressure overload or neurohormonal activation, triggers sustained inflammatory and pro-fibrotic signalling (*33*).

The fibrotic response can be divided into three phases: the initiative, the effective and the amplificative phase (*34*). Sustained activation by mechanical stress stimulates the production of circulating and myocardial cytokines and pro-fibrotic growth factors which (in the effective phase) bind to their receptors triggering the activation of transcriptional factors and signalling pathways resulting in the transformation of cardiac fibroblasts into myofibroblasts (*35*). Activated myofibroblasts synthesize excess collagen type I and III and expand the extracellular matrix (ECM) (*21*, *36*).

There are three main patterns of myocardial fibrosis – replacement, reactive interstitial and perivascular (Table 2). Replacement fibrosis reflects irreversible scarring after cardiomyocyte loss, while reactive interstitial and perivascular fibrosis represent diffuse remodelling that progresses despite preserved cardiomyocyte viability. Extensive fibrosis reduces the nutrient supply to the cardiomyocytes creating a vicious cycle of inflammation, cell death and ECM expansion (*37*).

**Table 2:** Types of myocardial fibrosis

**Type of fibrosis**	**Characteristics**	**Common cause**	**Functional impact**
Replacement	Collagen scar formation replacing cardiomyocytes Usually reparative and protective*	Myocardial infarction	Systolic dysfunction
Reactive interstitial	ECM accumulation without cardiomyocyte loss	Chronic activation of pro-fibrotic stimuli Hypertension, obesity, diabetes mellitus Microvascular dysfunction	Diastolic dysfunction Reduced left ventricular compliance Impaired myocardial perfusion
Perivascular	Expansion of microvascular adventitia		
*Replacement fibrosis has a critical protective role by ensuring the structural integrity of the heart and preventing mechanical complications after myocardial infarction (37). ECM - extracellular matrix.			

These tissue-level changes have an impact on diagnostic testing. Expansion of the ECM increases the distribution volume for gadolinium, affecting late gadolinium enhancement patterns and T1 extracellular volume mapping on CMR (*29*, *30*). Reduction of capillary density and alteration of mitochondrial function may impact tracer uptake in nuclear perfusion imaging, lowering the accuracy of PET and SPECT for viability assessment (*28*). Similarly, myocardial fibrosis limits contractile reserve, diminishing the sensitivity of DSE for identifying the potential for functional recovery (*15*).

Given the clinical significance of myocardial fibrosis, early detection and monitoring of its progression are crucial for improving patient outcomes. Circulating biomarkers of collagen turnover, fibrotic signalling and inflammation may serve as complementary tools and provide insight into myocardial remodelling.

## Biomarkers of myocardial fibrosis

Circulating biomarkers, detected in blood or urine, should ideally reflect the underlying pathophysiological processes in the target conditions or organs (*33*). The investigation of myocardial fibrosis biomarkers has received increasing attention in research communities. They are often molecules that play an essential part in the complex process of fibrosis development (*38*). Endomyocardial biopsy with quantification of collagen volume fraction by histology remains the gold standard for the diagnosis and staging of myocardial fibrosis. However, it is an invasive procedure with a risk of sampling error which limits its use to selected patients and research settings (*39*). This highlights the need for non-invasive imaging and circulating fibrosis biomarkers.

### Collagen synthesis biomarkers

As previously mentioned, myocardial fibrosis represents abnormal distribution and deposition of collagen, mainly increased collagen deposition and collagen turnover. Procollagen type I and III propeptides are markers of its synthesis and breakdown (*38*).

The ECM in the heart is composed predominantly of collagen type I (85%) and III (11%). They are both synthetized by cardiac fibroblasts as fibrillar collagen or procollagen, which is then split by proteinases in carboxy (C) and amino (N) terminal propeptides (*40*). Procollagen type I carboxy-terminal propeptide and procollagen type III amino-terminal propeptide are currently the only proven circulating peptides associated with proven myocardial interstitial fibrosis on endomyocardial biopsy (*41*).

Collagen type I is a fibrillar protein, aligned in fibres, present in almost all connective tissue structures, including bones, tendons, skin, sclera, blood vessels, as well as in other tissues providing a structural matrix. The dominant isoform is heterotrimer consisting of two α1 (I) and one α2 (I) chain (*17*). Its synthesis may be directly reflected by plasma concentrations of PICP because it is produced by cleavage in a ratio of 1:1 (*42*). On the other hand, PIIINP plasma concentrations may not be an accurate marker of collagen synthesis due to its smaller size, even though it has been linked to the amount of collagen type III fibres in the myocardium of heart failure patients (*43*).

Collagen type III is responsible for myocardial elasticity in reticular fibres in interstitial tissue of the heart, liver, lung and blood vessels. It is a homotrimer consisting of three α1 (III) chains overlapped in a triple helix (*17*). After proteases cleave propeptides of fibrillar collagen, PIIINP is released into the bloodstream by the lymphatics. It is considered to have an important role in fibril diameter, as well as having great storage capacity and stability (*42*).

A variety of immunoassay-based techniques are used to measure PICP and PIIINP, including enzyme-linked immunosorbent assays (ELISA), radioimmunoassays (RIA), and electrochemiluminescence immunoassays (ECLIA). However, there is considerable analytical variability between platforms due to differences in calibration standards, antibody specificity and sample preparation requirements. This variability complicates obtaining cross-study comparisons and universal cut-off value definitions (*43*). The preferred sample type is serum, as plasma anticoagulants and residual fibrinogen may introduce assay interference by interfering with antibody binding (*44*). Even in serum samples, lipids, fibrin and inadequate centrifugation can introduce measurement error, while storage conditions differ between research and routine clinical laboratories. These factors contribute to variation between different laboratories that may exceed normal biological differences between patient groups (*45*, *46*). Analytical performance and laboratory requirements of the most widely used assays for PICP and PIIINP are listed in Table 3 (*47*-*51*). While ECLIA-based methods show higher precision, greater automation and lower analytical variability, they are poorly harmonized across different manufacturers and reference ranges have not been standardized (*51*). On the other hand, ELISA kits are widely employed in research settings, but their higher inter-batch variability and operator-dependency limit clinical translation (*52*). Therefore, assay choice may impact study outcomes as much as underlying biological variation limiting the interpretation of PICP and PIIINP (*53*).

**Table 3:** Analytical performance and laboratory requirements of the most widely used assays for procollagen type I C-terminal propeptide (PICP) and procollagen type III N-terminal propeptide (PIIINP)

Assay*	**Method**	**Sample Type**	**Detection limit**	**Intra-assay variability**	**Inter-assay variability**	**Remarks^‡^**
Takara Bio PICP	Sandwich ELISA	Serum	~ 0.2 ng/mL	< 5%	< 7%	Manual ELISA; widely used in research, 96-well format
Orion Diagnostica UniQ PIIINP	Radioimmunoassay	Serum	~ 1 µg/L	~ 5%	~ 7%	Historical use; requires radiation license
General^†^	ECLIA	Serum	~ 0.1-0.5 ng/mL	< 3-5%	< 5-8%	Fully automated; high analytical precision; low sample volume; superior reproducibility
Cloud-Clone PICP	Competitive ELISA	Serum	~ 0.1 ng/mL	< 10%	< 12%	Research use only; limited clinical validation
MyBioSource PIIINP	Competitive ELISA	Serum	3.12 ng/ml	< 15%	< 15%	Research-grade; manual handling
*From (47–51). ^†^Performance values reflect the general analytical characteristics of ECLIA as standardized platform technology. Specific commercial ECLIA assays for PICP and PIIINP are not yet widely available. Values shown are based on validated ECLIA platforms reported in (51). ^‡^No standardized reference intervals or clinically validated cut-off values are currently available for circulating PICP or PIIINP as thresholds for myocardial viability or revascularization decision-making; reported values in the literature are assay- and disease-specific. ELISA - enzyme-linked immunosorbent assay. ECLIA - electrochemiluminescence immunoassay.						

Concentrations of PIIINP are positively correlated with diastolic dysfunction in patients with heart failure with reduced ejection fraction, as well as left ventricular mass index and relative wall thickness in patients with left ventricular hypertrophy who have undergone successful coarctation of the aorta repair (*54*, *55*). A cross-sectional study conducted by Yang *et al.* demonstrated that in patients with hypertrophic cardiomyopathy, plasma PICP concentrations correlated with myocardial PICP content and myocardial collagen volume fraction on histology (*56*). Ferreira *et al.* showed that PICP concentrations were significantly higher in hypertensive patients before treatment (*57*).

In the study by Raafs *et al.,* fibrosis was quantified in 209 patients with dilated cardiomyopathy using endomyocardial biopsy with determination of collagen volume fraction, CMR with late gadolinium enhancement and circulating PICP and PIIINP concentrations (*58*). They found that circulating PICP concentrations were significantly higher in patients with myocardial fibrosis on CMR (91 (67–112) ng/mL *vs.* 77 (62–97) ng/mL, P = 0.02). RNA sequencing of endomyocardial biopsy tissue confirmed the increased expression of pro-inflammatory and pro-fibrotic pathways in patients with elevated PICP concentrations, and also demonstrated significant correlation with histologically proven myocardial fibrosis (R^2^ = 0.17, P = 0.001). Furthermore, PICP was independently associated with adverse cardiovascular events and mortality. They did not find such association or correlation with PIIINP (*58*).

In patients with non-ischemic dilated cardiomyopathy, elevated serum concentrations of PICP and PIIINP were associated with CMR findings of myocardial fibrosis (156 ng/mL *vs.* 74 ng/mL, P < 0.001; and 5.1 ng/mL *vs.* 3.5 ng/mL, P < 0.001, respectively). Additionally, a cut-off value of 44.4 ng/mL for PICP predicted myocardial fibrosis with 77.5% sensitivity, 76% specificity and a negative predictive value of 85.5%, while PIIINP at a cut-off value of 1.18 ng/mL had a 71.83% sensitivity, 83% specificity and a negative predictive value of 83.6% (*59*). However, these cut-off values were derived from a single disease and cannot be assumed for ischemic diseases or viability assessment due to different underlying mechanisms. No universally accepted diagnostic cut-offs exist for PICP or PIIINP; their values currently remain disease dependent and cannot be applied as criteria for revascularization.

The clinical diagnostic utility of PICP and PIIINP was investigated in a systematic review and meta-analysis conducted by Zhang *et al.* (*60*). They reviewed 1130 records from four databases and included 12 studies after independent screening. The results confirmed that patients with myocardial fibrosis had significantly elevated serum PICP (95% confidence interval (CI) = 0.40 to 1.40) and PIIINP (95% CI = 0.04 to 1.23) (*60*). A study by Ravassa *et al.* explored utility of PICP in differentiating patients with heart failure who would be more likely to experience myocardial recovery. They found that patients with lower PICP (< 108.1 ng/mL) showed greater left ventricular reverse remodelling and lower risk of outcome related to heart failure (*61*).

Patient characteristics such as age, body mass index, comorbidities and heart failure treatment (particularly spironolactone) can alter concentrations of these biomarkers by their effect on collagen turnover (*62*). It has been suggested that propeptides may be incorporated in the collagen fibre network, preventing them from being cleaved leading to underestimation of true PICP and PIIINP concentrations even in patients with extensive myocardial fibrosis (*63*). Also, circulating concentrations reflect systemic fibrosis; elevated concentrations may originate from non-cardiac fibrosis involving the liver, kidneys, bones or lungs. Therefore, increased concentration does not necessarily reflect *in situ* myocardial fibrosis (*40*). This population-dependent variability and lack of tissue specificity negatively impact the ability of PICP and PIIINP to discriminate reversible from irreversible myocardial dysfunction which is required to guide revascularization decisions.

### Galectin-3

Galectin-3 regulates several cellular functions: growth, differentiation, proliferation, adhesion, apoptosis and tissue repair (*64*). Most commonly, it is located in the cytoplasm, as well as being expressed on the cellular surface. It is then secreted into biological fluids such as urine and blood. Additionally, injured and inflammatory cells release it under different pathological conditions (*18*). Galectin-3 is a potent inflammatory protein involved in acute and chronic inflammation by initiating and amplifying the inflammatory response (*65*, *66*).

It is measured in plasma or serum using immunoassay-based techniques, most commonly ELISA or ECLIA (*67*). Galectin-3 concentrations do not differ significantly when measured in serum or plasma (*68*). Commercially available assays have different sensitivity and specificity, with reported limits of detection typically ranging from 0.1-0.3 ng/mL. Intra- and inter-assay coefficients of variation of different platforms are typically under 10% which is acceptable for clinical use, although platform-specific variability remains a major limitation to clinical interpretation (*69*). Consequently, Gal-3 concentrations cannot be directly compared across studies unless the same assay platform is used, while the absence of cross-platform standardization prevents establishment of universal cut-off values. Therefore, reported Gal-3 normal ranges cannot be uniformly applied and assay-specific interpretation remains necessary.

A study on rat models after myocardial infarction showed increased concentrations of Gal-3 and a later peak in non-infarcted myocardium demonstrating its role in cardiac remodelling (*70*). In a study by Liu *et al.*, they infused Gal-3 into rats intrapericardially and reported its overexpression compromising the cardiomyocyte´s viability. They also noted elevated mast cell and macrophage infiltration, increased perivascular and interstitial fibrosis and cardiac hypertrophy (*71*).

Expression of Gal-3 is associated with increased fibroblast activity, ECM accumulation and production of collagen in the myocardium. It is also expressed in fibroblasts and macrophages after stressful events (*72*, *73*). After activation, it forms a complex with transforming growth factor beta on the cell surface which stimulates fibrosis development. This signal, along with mechanical stress, transforms fibroblasts into active myofibroblasts that produce collagen (*74*).

Some recent studies investigated the Gal-3 upper reference limit in a healthy population of blood donors. They measured Gal-3 by using the Architect STAT Galectin-3 immunoassay. Median Gal-3 plasma concentration was 14.3 ng/mL (interquartile range 11.9-16.7 ng/mL), while the 97.5th percentile upper reference limit (URL) of normal in their study population (90% CI) was 26.1 (23.3-31.5) ng/mL. No sex-related differences were found. In contrast, age was a confounding variable that affected its concentration – the URL of Gal-3 was found to be higher in older (> 45 years) than in younger subjects (31.5 (26.2-51.4) *vs.* 21.8 (21-26.1) ng/mL, respectively) (*75*, *76*). This indicates that population-based reference intervals are affected by age, as well as being assay-specific, limiting their routine clinical interpretation.

In a study of patients with non-ischemic dilated cardiomyopathy, elevated Gal-3 concentrations were associated with findings of myocardial fibrosis on CMR (17.7 ng/mL *vs*. 9.1 ng/mL, P < 0.001). Furthermore, a cut-off value of 11 ng/mL predicted myocardial fibrosis with 90.4% sensitivity, 66.1% specificity and 92% negative predictive value (*59*). These thresholds were derived from a single disease entity, which cannot be anticipated for viability assessment in ischemic disease due to different underlying mechanisms. For example, remodelling after myocardial infarction involves acute macrophage activation and transient sharp Gal-3 elevation, while chronic ischemic disease is characterized by lower inflammatory markers as part of chronic interstitial fibrosis. Consequently, similar Gal-3 concentrations may reflect transient and reversible inflammation in certain patients but irreversible fibrosis in others, depending on the remodelling mechanism (*64*, *77*).

Galectin-3 measurement is endorsed by the 2017 Guidelines of the American Heart Association for assessing risk and evaluating prognosis of patients with heart failure. Different mechanisms are involved in the promotion of heart failure by Gal-3, some of which are: inflammatory cell infiltration, fibroblast proliferation and cardiomyocytes hypertrophy (*64*). The threshold of 17.8 ng/mL is often considered to successfully discriminate between low-risk and high-risk for clinical complications in heart failure patients (*74*).

Galectin-3 has also been investigated in other cardiovascular diseases, especially those initiated and stimulated by inflammation, where elevated concentrations reflect disease activity and severity, as well as adverse prognosis (*78*-*82*). In a study by Screever *et al.* they investigated the association of CMR-identified fibrosis with Gal-3 after myocardial infarction. Concentrations of Gal-3 were higher in patients with CMR-identified fibrosis (20 *vs.* 15 ng/mL, P = 0.004) (*83*). In another study by Asleh *et al*., Gal-3 concentrations above 15.1 ng/mL were associated with a higher risk of heart failure and death after myocardial infarction, even after adjustment for age, sex, comorbidities and troponin levels (*84*). A study by Sherpa *et al.* showed that elevated concentrations of Gal-3 are associated with a higher risk of myocardial fibrosis and sudden cardiac death (*85*). This shows the importance of larger studies that would target Gal-3 to prevent myocardial fibrosis and lower the risk of sudden cardiac death.

### Limitations and clinical utility of fibrosis biomarkers in predicting myocardial viability

Even though circulating biomarkers such as PICP, PIIINP and Gal-3 reflect remodelling of the ECM, they have rarely been studied in association with functional recovery after revascularization, the clinical definition of myocardial viability. Published data mostly investigate their correlation with fibrosis burden on non-invasive imaging, without an evaluation of contractility improvement, perfusion or symptom relief after revascularization (*43*, *63*). Additionally, elevated circulating biomarker concentrations do not indicate irreversible scar, as they may also reflect active inflammation or diffuse interstitial fibrosis without loss of function (*64*). Different concentrations across various cardiovascular diseases, limited tissue specificity and lack of standardized cut-offs reduce their discriminative power in distinguishing reversible from irreversible myocardial dysfunction. There is currently no biomarker cut-off that reliably differentiates myocardium capable of recovery from an irreversible scar.

They provide information on underlying biological processes of myocardial remodelling, but currently lack outcome-based validation. Consequently, their use remains primarily experimental. They should be interpreted as complementary indicators of remodelling, not as the determinants of revascularization decisions. Well-designed clinical studies incorporating biomarkers with contemporary imaging are required before their usage in routine viability assessment.

To enable comparison of the available circulating fibrosis biomarkers, Table 4 summarizes their underlying biological pathways, commonly used analytical methods, current level of clinical validation and key strengths and limitations.

**Table 4:** Comparison of circulating fibrosis biomarkers relevant to myocardial viability assessment

**Biomarker**	**Biological pathway**	**Analytical methods**	**Clinical validation**	**Strengths**	**Limitations**
PICP	Collagen type I synthesis Replacement and interstitial fibrosis	ELISA RIA ECLIA	Moderate Correlates with CMR fibrosis, biopsy collagen volume fraction, remodelling and outcomes in heart failure Limited data in ischemic viability	Strongest correlation with collagen type I turnover Automated ECLIA platforms available Independent association with adverse outcomes	Affected by systemic fibrosis (bone, liver) Assay variability and lack of harmonized cut-offs Underestimates fibrosis if propeptides remain in extracellular matrix
PIIINP	Collagen type III synthesis Early interstitial fibrosis	ELISA RIA ECLIA	Low–moderate Associated with heart failure severity and remodelling	Reflects dynamic collagen turnover Useful in heart failure and hypertrophic cardiomyopathy	Less specific for myocardial fibrosis Higher biological variability Influenced by liver disease, obesity, systemic inflammation Limited prognostic value
Galectin-3	Macrophage activation Inflammation Fibrosis signalling Fibroblast proliferation	ELISA ECLIA	Moderate Validated predictor of heart failure hospitalization and mortality Correlates with diffuse fibrosis on CMR	Reflects upstream fibrotic signalling Stable in serum/plasma Large body of clinical outcome data	Not specific to cardiac tissue Influenced by age and renal function Assay variability and non-unified reference intervals Elevations may reflect inflammation
PICP - procollagen type I C-terminal propeptide. PIIINP - procollagen type III N-terminal propeptide. ELISA - enzyme-linked immunosorbent assay. RIA - radioimmunoassay. ECLIA - electrochemiluminescence immunoassay. CMR - cardiac magnetic resonance.					

### Telomere length determination as an emerging biomarker of myocardial fibrosis

Cellular aging and repeated inflammatory or oxidative stress progressively shorten the telomeres. These processes are also integral to fibroblast activation and ECM expansion (*86*). Studies have shown that shortened leukocyte telomeres correlate with the degree of cardiac aging (*87*).

Telomere length can be measured with fluorescence in-situ hybridization (flow-FISH), quantitative polymerase chain reaction (PCR) or Southern blot analysis, with quantitative PCR being the most widely used because of its low sample requirements and scalability. Nevertheless, lack of standardized reference ranges, assay variability and low specificity currently limit its clinical application (*88*).

Despite these limitations, telomere length assessment provides insight into the burden of remodelling and cellular stress. In future, it may complement other biomarkers to improve stratification of patients with chronic coronary syndrome, especially those with CTO in whom the degree of irreversible remodelling influences viability and benefit from revascularization.

## Conclusion

Reliable myocardial viability evaluation represents a key step in selecting patients with chronic coronary syndrome and CTO who are most likely to benefit from revascularization. Circulating fibrosis biomarkers, such as PICP, PIIINP and Gal-3 reflect ECM remodelling and may complement non-invasive imaging modalities in identifying patients with irreversible myocardial fibrosis who are less likely to benefit from revascularization. However, these biomarkers are currently associated with fibrosis burden rather than functional recovery after revascularization. No biomarker cut-off has been prospectively validated in large-scale studies to reliably discriminate reversible dysfunction from irreversible scar.

Circulating fibrosis biomarkers should therefore be used as adjunctive tools that reflect underlying biological processes, not as standalone guides for revascularization decisions. Their integration into clinical guidelines and pathways requires large-scale prospective outcome-based studies that link biomarker concentrations with contractility recovery and perfusion improvement. Future research should focus on standardizing assays and reference intervals, finding disease-specific cut-offs that differentiate acute inflammation from established fibrosis, as well as prospective strategies that combine biomarkers with advanced non-invasive imaging modalities.

## Data Availability

No data was generated during this study, so data sharing statement is not applicable to this article.

## References

[1] Ybarra LF Rinfret S Brilakis ES Karmpaliotis D Azzalini L Grantham JA Definitions and clinical trial design principles for coronary artery chronic total occlusion therapies: CTO-ARC Consensus Recommendations. Circulation. 2021;143:479–500. 10.1161/CIRCULATIONAHA.120.046754 33523728

[2] Azzalini L Jolicoeur EM Pighi M Millán X Picard F Tadros VX Epidemiology, management strategies, and outcomes of patients with chronic total coronary occlusion. Am J Cardiol. 2016;118:1128–35. 10.1016/j.amjcard.2016.07.023 27561190

[3] Simsek B Kostantinis S Karacsonyi J Alaswad K Krestyaninov O Khelimskii D Predicting periprocedural complications in chronic total occlusion percutaneous coronary intervention: The PROGRESS-CTO complication scores. JACC Cardiovasc Interv. 2022;15:1413–22. 10.1016/j.jcin.2022.06.007 35863789

[4] Koelbl CO Nedeljkovic ZS Jacobs AK. Coronary chronic total occlusion (CTO): A review. Rev Cardiovasc Med. 2018;19:33–9. 10.31083/j.rcm.2018.01.896 31032601

[5] Steg PG Greenlaw N Tendera M Tardif JC Ferrari R Al-Zaibag M Prevalence of anginal symptoms and myocardial ischemia and their effect on clinical outcomes in outpatients with stable coronary artery disease: Data from the international observational CLARIFY registry. JAMA Intern Med. 2014;174:1651–9. 10.1001/jamainternmed.2014.3773 25110899

[6] Brilakis ES Banerjee S Karmpaliotis D Lombardi WL Tsai TT Shunk KA Procedural outcomes of chronic total occlusion percutaneous coronary intervention: A report from the NCDR (National Cardiovascular Data Registry). JACC Cardiovasc Interv. 2015;8:245–53. 10.1016/j.jcin.2014.08.014 25700746

[7] Galassi AR Boukhris M Toma A Elhadj ZI Laroussi L Gaemperli O Percutaneous coronary intervention of chronic total occlusions in patients with low left ventricular ejection fraction. JACC Cardiovasc Interv. 2017;10:2158–70. 10.1016/j.jcin.2017.06.058 29055762

[8] Yeh RW Tamez H Secemsky EA Grantham JA Sapontis J Spertus JA Depression and angina among patients undergoing chronic total occlusion percutaneous coronary intervention: The OPEN-CTO registry. JACC Cardiovasc Interv. 2019;12:651–8. 10.1016/j.jcin.2018.12.029 30878475

[9] Obedinskiy AA Kretov EI Boukhris M Kurbatov VP Osiev AG Ibn Elhadj Z The IMPACTOR-CTO trial. JACC Cardiovasc Interv. 2018;11:1309–11. 10.1016/j.jcin.2018.04.017 29976368

[10] Juricic SA Tesic MB Galassi AR Petrovic ON Dobric MR Orlic DN Randomized controlled comparison of optimal medical therapy with percutaneous recanalization of chronic total occlusion (COMET-CTO). Int Heart J. 2021;62:16–22. 10.1536/ihj.20-427 33518655

[11] Werner GS Martin-Yuste V Hildick-Smith D Boudou N Sianos G Gelev V A randomized multicentre trial to compare revascularization with optimal medical therapy for the treatment of chronic total coronary occlusions. Eur Heart J. 2018;39:2484–93. 10.1093/eurheartj/ehy220 29722796

[12] Elias J Van Dongen IM Hoebers LP Ouweneel DM Claessen BEPM Råmunddal T Improved recovery of regional left ventricular function after PCI of chronic total occlusion in STEMI patients: a cardiovascular magnetic resonance study of the randomized controlled EXPLORE trial. J Cardiovasc Magn Reson. 2017;19:53. 10.1186/s12968-017-0369-z 28724418 PMC5517806

[13] Megaly M Saad M Tajti P Burke MN Chavez I Gössl M Meta-analysis of the impact of successful chronic total occlusion percutaneous coronary intervention on left ventricular systolic function and reverse remodeling. J Interv Cardiol. 2018;31:562–71. 10.1111/joic.12538 29974508

[14] Canton L Suma N Amicone S Impellizzeri A Bodega F Marinelli V Clinical impact of multimodality assessment of myocardial viability. Echocardiography. 2024;41:e15854. 10.1111/echo.15854 38940225

[15] Garcia MJ Kwong RY Scherrer-Crosbie M Taub CC Blankstein R Lima J State of the art: Imaging for myocardial viability: A scientific statement from the American Heart Association. Circ Cardiovasc Imaging. 2020;13:e000053. 10.1161/HCI.0000000000000053 32833510

[16] Bing R Dweck MR. Myocardial fibrosis: why image, how to image and clinical implications. Heart. 2019;105:1832–40. 10.1136/heartjnl-2019-315560 31649047 PMC6900237

[17] Nikolov A Popovski N. Extracellular matrix in heart disease: Focus on circulating collagen type i and iii derived peptides as biomarkers of myocardial fibrosis and their potential in the prognosis of heart failure: A concise review. Metabolites. 2022;12:297. 10.3390/metabo12040297 35448484 PMC9025448

[18] Hara A Niwa M Noguchi K Kanayama T Niwa A Matsuo M Galectin-3 as a next-generation biomarker for detecting early stage of various diseases. Biomolecules. 2020;10:389. 10.3390/biom10030389 32138174 PMC7175224

[19] Ryan M Morgan H Chiribiri A Nagel E Cleland J Perera D. Myocardial viability testing: all STICHed up, or about to be REVIVED? Eur Heart J. 2022;43:118. 10.1093/eurheartj/ehab729 34791132 PMC8757581

[20] Vaidya Y, Cavanaugh SM, Dhamoon AS. Myocardial stunning and hibernation. StatPearls [Internet]. 2023 Aug [cited 2024 Nov 3]; Available from: https://www.ncbi.nlm.nih.gov/books/NBK537026/. 30725711

[21] Bengel FM Diekmann J Hess A Jerosch-Herold M. Myocardial fibrosis: Emerging target for cardiac molecular imaging and opportunity for image-guided therapy. J Nucl Med. 2023;64:49S–58S. 10.2967/jnumed.122.264867 37918842

[22] Babes EE Tit DM Bungau AF Bustea C Rus M Bungau SG Myocardial viability testing in the management of ischemic heart failure. Life (Basel). 2022;12:1760. 10.3390/life12111760 36362914 PMC9698475

[23] Li DL Kronenberg MW. Myocardial perfusion and viability imaging in coronary artery disease: clinical value in diagnosis, prognosis, and therapeutic guidance. Am J Med. 2021;134:968–75. 10.1016/j.amjmed.2021.03.011 33864764

[24] Madsen S Dias AH Lauritsen KM Bouchelouche K Tolbod LP Gormsen LC. Myocardial viability testing by positron emission tomography: Basic concepts, mini-review of the literature and experience from a tertiary PET center. Semin Nucl Med. 2020;50:248–59. 10.1053/j.semnuclmed.2020.02.010 32284111

[25] Lee Y Jang J Lim S Na SJ. Evaluation of clinical variables affecting myocardial glucose uptake in cardiac FDG PET. Diagnostics (Basel). 2024;14:1705. 10.3390/diagnostics14161705 39202193 PMC11353438

[26] Singh V Dorbala S. Normal variants and pitfalls in cardiac PET/CT. Semin Nucl Med. 2021;51:441–57. 10.1053/j.semnuclmed.2021.04.004 34049686

[27] Manapragada PP Andrikopoulou E Bajaj N Bhambhvani P. PET cardiac imaging (perfusion, viability, sarcoidosis, and infection). Radiol Clin North Am. 2021;59:835–52. 10.1016/j.rcl.2021.05.009 34392922

[28] Katikireddy CK Samim A. Myocardial viability assessment and utility in contemporary management of ischemic cardiomyopathy. Clin Cardiol. 2022;45:152–61. 10.1002/clc.23779 35077580 PMC8860488

[29] Sohn SH Kang Y Kim JS Park EA Lee W Hwang HY. Impact of myocardial viability on long-term outcomes after surgical revascularization. Thorac Cardiovasc Surg. 2024;72:441–8. 10.1055/a-2228-7104 38092064

[30] Yang X Tian J Zhang L Dong W Mi H Li J Myocardial viability, functional status, and collaterals of patients with chronically occluded coronary arteries. Front Cardiovasc Med. 2021;8:754826. 10.3389/fcvm.2021.754826 34869665 PMC8632801

[31] Travers JG Kamal FA Robbins J Yutzey KE Blaxall BC. Cardiac fibrosis: The fibroblast awakens. Circ Res. 2016;118:1021–40. 10.1161/CIRCRESAHA.115.306565 26987915 PMC4800485

[32] Ma ZG Yuan YP Wu HM Zhang X Tang QZ. Cardiac fibrosis: new insights into the pathogenesis. Int J Biol Sci. 2018;14:1645. 10.7150/ijbs.28103 30416379 PMC6216032

[33] Suthahar N Meijers WC Silljé HHW de Boer RA. From inflammation to fibrosis-molecular and cellular mechanisms of myocardial tissue remodelling and perspectives on differential treatment opportunities. Curr Heart Fail Rep. 2017;14:235–50. 10.1007/s11897-017-0343-y 28707261 PMC5527069

[34] Kong P Christia P Frangogiannis NG. The pathogenesis of cardiac fibrosis. Cell Mol Life Sci. 2014;71:549–74. 10.1007/s00018-013-1349-6 23649149 PMC3769482

[35] Saadat S Noureddini M Mahjoubin-Tehran M Nazemi S Shojaie L Aschner M Pivotal role of TGF-β/Smad signaling in cardiac fibrosis: Non-coding RNAs as effectual players. Front Cardiovasc Med. 2021;7:588347. 10.3389/fcvm.2020.588347 33569393 PMC7868343

[36] Frangogiannis NG. Cardiac fibrosis. Cardiovasc Res. 2021;117:1450–88. 10.1093/cvr/cvaa324 33135058 PMC8152700

[37] Piek A de Boer RA Silljé HHW. The fibrosis-cell death axis in heart failure. Heart Fail Rev. 2016;21:199–211. 10.1007/s10741-016-9536-9 26883434 PMC4762920

[38] An Z Yang G Zheng H Nie W Liu G. Biomarkers in patients with myocardial fibrosis. Open Life Sci. 2017;12:337–44. 10.1515/biol-2017-0039

[39] Hahn VS Yanek LR Vaishnav J Ying W Vaidya D Lee YZJ Endomyocardial biopsy characterization of heart failure with preserved ejection fraction and prevalence of cardiac amyloidosis. JACC Heart Fail. 2020;8:712–24. 10.1016/j.jchf.2020.04.007 32653448 PMC7604801

[40] Lijnen PJ Maharani T Finahari NS Prihadi J. Serum collagen markers and heart failure. Cardiovasc Hematol Disord Drug Targets. 2012;12:51–5. 10.2174/187152912801823147 22524171

[41] González A Schelbert EB Díez J Butler J. Myocardial interstitial fibrosis in heart failure: biological and translational perspectives. J Am Coll Cardiol. 2018;71:1696–706. 10.1016/j.jacc.2018.02.021 29650126

[42] Ding Y Wang Y Zhang W Jia Q Wang X Li Y Roles of biomarkers in myocardial fibrosis. Aging Dis. 2020;11:1157–74. 10.14336/AD.2020.0604 33014530 PMC7505259

[43] López B González A Ravassa S Beaumont J Moreno MU San José G Circulating biomarkers of myocardial fibrosis: The need for a reappraisal. J Am Coll Cardiol. 2015;65:2449–56. 10.1016/j.jacc.2015.04.026 26046739

[44] Bolton JS Chaudhury S Dutta S Gregory S Locke E Pierson T Comparison of ELISA with electro-chemiluminescence technology for the qualitative and quantitative assessment of serological responses to vaccination. Malar J. 2020;19:159. 10.1186/s12936-020-03225-5 32303235 PMC7165447

[45] Lippi G Cadamuro J Von Meyer A Simundic AM. Practical recommendations for managing hemolyzed samples in clinical chemistry testing. Clin Chem Lab Med. 2018;56:718–27. 10.1515/cclm-2017-1104 29373316

[46] Tuck MK Chan DW Chia D Godwin AK Grizzle WE Krueger KE Standard operating procedures for serum and plasma collection: Early detection research network consensus statement standard operating procedure integration working group. J Proteome Res. 2009;8:113. 10.1021/pr800545q 19072545 PMC2655764

[47] ELISA Kit for procollagen i c-terminal propeptide (PICP) | SEA570Hu | Homo sapiens (Human) CLOUD-CLONE CORP.(CCC). Available from: https://www.cloud-clone.com/products/SEA570Hu.html. Accessed July 21st 2025.

[48] Ordering information technical information. Available from: www.oriondiagnostica.com. Accessed July 21st 2025.

[49] Procollagen type I C-peptide (PIP) detection. Available from: https://www.takarabio.com/products/antibodies-and-elisa/primary-antibodies-and-elisas-by-research-area/bone-research/procollagen-type-i-c-peptide?srsltid=AfmBOorB-PVz2IEMJ6dZ0ODHaOiKc5dlptpNtexlqpvO9t9HQ9owdrhz. Accessed July 21st 2025.

[50] PIIINP elisa kit | Human Procollagen III N-Terminal Propeptide ELISA Kit. Available from: https://www.mybiosource.com/piiinp-human-elisa-kits/procollagen-iii-n-terminal-propeptide/76351. Accessed July 21st 2025.

[51] Premnath SM, Zubair M. Electrochemiluminescence method. StatPearls [Internet]. 2023 Jun [cited 2025 Jul 21]; Available from: https://www.ncbi.nlm.nih.gov/books/NBK594228/.

[52] Aydin S Emre E Ugur K Aydin MA Sahin İ Cinar V An overview of ELISA: a review and update on best laboratory practices for quantifying peptides and proteins in biological fluids. J Int Med Res. 2025;53:3000605251315913. 10.1177/03000605251315913 39922798 PMC11808753

[53] Rubiś PP Dziewięcka E González A Cleland JGF. High variability in assays of blood markers of collagen turnover in cardiovascular disease: Implications for research and clinical practice. Eur J Heart Fail. 2025;27:901–4. 10.1002/ejhf.3375 38980205

[54] Lin YT Lin YH Wu XM Ko CL Yen RF Chen YH The relationship between serum fibrosis markers and restrictive ventricular filling in patients with heart failure with reduced ejection fraction: A technetium-99m radionuclide ventriculography study. Oncotarget. 2017;8:2381–90. 10.18632/oncotarget.13795 27924061 PMC5356808

[55] Yamazawa H Murakami T Takeda A Takei K Furukawa T Nakajima H. Serum concentration of procollagen type III amino-terminal peptide is increased in patients with successfully repaired coarctation of the aorta with left ventricular hypertrophy. Pediatr Cardiol. 2015;36:555–60. 10.1007/s00246-014-1049-5 25311763

[56] Yang C Qiao S Song Y Liu Y Tang Y Deng L Procollagen type I carboxy-terminal propeptide (PICP) and MMP-2 are potential biomarkers of myocardial fibrosis in patients with hypertrophic cardiomyopathy. Cardiovasc Pathol. 2019;43:107150. 10.1016/j.carpath.2019.107150 31639652

[57] Ferreira JP Rossignol P Pizard A Machu JL Collier T Girerd N Potential spironolactone effects on collagen metabolism biomarkers in patients with uncontrolled blood pressure. Heart. 2019;105:307–14. 10.1136/heartjnl-2018-313182 30121630

[58] Raafs AG Verdonschot JAJ Henkens MTHM Adriaans BP Wang P Derks K The combination of carboxy-terminal propeptide of procollagen type I blood levels and late gadolinium enhancement at cardiac magnetic resonance provides additional prognostic information in idiopathic dilated cardiomyopathy - A multilevel assessment of myocardial fibrosis in dilated cardiomyopathy. Eur J Heart Fail. 2021;23:933–44. 10.1002/ejhf.2201 33928704 PMC8362085

[59] Revnic R Cojan-Minzat BO Zlibut A Orzan RI Agoston R Muresan ID The role of circulating collagen turnover biomarkers and late gadolinium enhancement in patients with non-ischemic dilated cardiomyopathy. Diagnostics (Basel). 2022;12:1435. 10.3390/diagnostics12061435 35741245 PMC9222171

[60] Zhang T Xue Q Zhu R Jiang Y. Diagnostic value of PICP and PIIINP in myocardial fibrosis: A systematic review and meta-analysis. Clin Cardiol. 2022. 10.1002/clc.23901 36468277

[61] Ravassa S Lupón J López B Codina P Domingo M Revuelta-López E Prediction of left ventricular reverse remodeling and outcomes by circulating collagen-derived peptides. JACC Heart Fail. 2023;11:58–72. 10.1016/j.jchf.2022.09.008 36599551

[62] Pellicori P Ferreira JP Mariottoni B Brunner-La Rocca HP Ahmed FZ Verdonschot J Effects of spironolactone on serum markers of fibrosis in people at high risk of developing heart failure: rationale, design and baseline characteristics of a proof-of-concept, randomised, precision-medicine, prevention trial. The Heart OMics in AGing (HOMAGE) trial. Eur J Heart Fail. 2020;22:1711–23. 10.1002/ejhf.1716 31950604

[63] Nikolov A Tzekova M Kostov K. Serum biomarkers of collagen type i and type iii turnover in heart failure – the need for reappraisal. Acta Med Croatica. 2020;74:145–52.

[64] Seropian IM Cassaglia P Miksztowicz V González GE. Unraveling the role of Galectin-3 in cardiac pathology and physiology. Front Physiol. 2023;14:1304735. 10.3389/fphys.2023.1304735 38170009 PMC10759241

[65] Wang X Gaur M Mounzih K Rodriguez HJ Qiu H Chen M Inhibition of Galectin-3 post-infarction impedes progressive fibrosis by regulating inflammatory profibrotic cascades. Cardiovasc Res. 2023;119:2536–49. 10.1093/cvr/cvad116 37602717 PMC10676456

[66] Bošnjak I Selthofer-Relatic K Včev A. Prognostic value of galectin-3 in patients with heart failure. Dis Markers. 2015;2015:690205. 10.1155/2015/690205 25960597 PMC4415488

[67] de Boer RA Lok DJA Jaarsma T Van Der Meer P Voors AA Hillege HL Predictive value of plasma galectin-3 levels in heart failure with reduced and preserved ejection fraction. Ann Med. 2011;43:60–8. 10.3109/07853890.2010.538080 21189092 PMC3028573

[68] Christenson RH Duh SH Wu AHB Smith A Abel G deFilippi CR Multi-center determination of Galectin-3 assay performance characteristics: Anatomy of a novel assay for use in heart failure. Clin Biochem. 2010;43:683–90. 10.1016/j.clinbiochem.2010.02.001 20153309

[69] Lok DJA Van Der Meer P De La Porte PWBA Lipsic E Van Wijngaarden J Hillege HL Prognostic value of Galectin-3, a novel marker of fibrosis in patients with chronic heart failure: Data from the DEAL-HF study. Clin Res Cardiol. 2010;99:323–8. 10.1007/s00392-010-0125-y 20130888 PMC2858799

[70] Sanchez-Mas J Lax A Asensio-Lopez MC Fernandez-Del Palacio MJ Caballero L Garrido IP Galectin-3 expression in cardiac remodeling after myocardial infarction. Int J Cardiol. 2014;172:e98–101. 10.1016/j.ijcard.2013.12.129 24433619

[71] Liu YH D’Ambrosio M Liao TD Peng H Rhaleb NE Sharma U N-acetyl-seryl-aspartyl-lysyl-proline prevents cardiac remodeling and dysfunction induced by Galectin-3, a mammalian adhesion/growth-regulatory lectin. Am J Physiol Heart Circ Physiol. 2009;296:H404–12. 10.1152/ajpheart.00747.2008 19098114 PMC2643891

[72] Bouffette S Botez I De Ceuninck F. Targeting Galectin-3 in inflammatory and fibrotic diseases. Trends Pharmacol Sci. 2023;44:519–31. 10.1016/j.tips.2023.06.001 37391294

[73] Seropian IM El-Diasty M El-Sherbini AH González GE Rabinovich GA. Central role of Galectin-3 at the cross-roads of cardiac inflammation and fibrosis: Implications for heart failure and transplantation. Cytokine Growth Factor Rev. 2024;80:47–58. 10.1016/j.cytogfr.2024.10.002 39482190

[74] Blanda V Bracale UM Di Taranto MD Fortunato G. Galectin-3 in cardiovascular diseases. Int J Mol Sci. 2020;21:1–18. 10.3390/ijms21239232 33287402 PMC7731136

[75] Agnello L Bellia C Lo Sasso B Pivetti A Muratore M Scazzone C Establishing the upper reference limit of Galectin-3 in healthy blood donors. Biochem Med (Zagreb). 2017;27:030709. 10.11613/BM.2017.030709 29180917 PMC5696745

[76] Mueller T Egger M Leitner I Gabriel C Haltmayer M Dieplinger B. Reference values of Galectin-3 and cardiac troponins derived from a single cohort of healthy blood donors. Clin Chim Acta. 2016;456:19–23. 10.1016/j.cca.2016.02.014 26920637

[77] Leancă SA Crișu D Petriș AO Afrăsânie I Genes A Costache AD Left ventricular remodeling after myocardial infarction: From physiopathology to treatment. Life. 2022;12:1111. 10.3390/life12081111 35892913 PMC9332014

[78] Lyngbakken MN Myhre PL Røsjø H Omland T. Novel biomarkers of cardiovascular disease: Applications in clinical practice. Crit Rev Clin Lab Sci. 2019;56:33–60. 10.1080/10408363.2018.1525335 30457415

[79] Bošnjak I Bedeković D. Selthofer- Relatić K, Bilić-Ćurčić I. Galectin-3: A heart failure biomarker as sign of active coronary heart disease. World J Cardiovasc Dis. 2017;7:373–9. 10.4236/wjcd.2017.711035

[80] Bošnjak I Bedeković D Selthofer-Relatić K Roguljić H Bilić-Ćurčić I. Association of galectin-3 and significant atherosclerotic epicardial artery disease in patients with chronic coronary syndrome. Cardiol Croat. 2022;17:167. 10.15836/ccar2022.167

[81] Bošnjak I Bedeković D Selthofer-Relatić K Roguljić H Mihaljević I Dukić D Heart failure biomarkers in revascularized patients with stable coronary heart disease as clinical outcome predictors. Front Cardiovasc Med. 2024;11:1458120. 10.3389/fcvm.2024.1458120 39346100 PMC11428046

[82] Bošnjak I Bedeković D Selthofer-Relatić K Roguljić H Mihaljević I Bilić-Ćurčić I. Role of galectin-3 in diagnosis and severity assessment of epicardial artery lesions in patients with suspected coronary artery disease. BMC Cardiovasc Disord. 2023;23:268. 10.1186/s12872-023-03310-y 37221462 PMC10204313

[83] Screever EM Gorter TM Willems TP Aboumsallem JP Suthahar N Mahmoud B Diffuse myocardial fibrosis on cardiac magnetic resonance imaging is related to Galectin-3 and predicts outcome in heart failure. Biomolecules. 2023;13:410. 10.3390/biom13030410 36979345 PMC10046101

[84] Asleh R Enriquez-Sarano M Jaffe AS Manemann SM Weston SA Jiang R Galectin-3 levels and outcomes after myocardial infarction: A population-based study. J Am Coll Cardiol. 2019;73:2286–95. 10.1016/j.jacc.2019.02.046 31072572 PMC6512841

[85] Sherpa MD Sonkawade SD Jonnala V Pokharel S Khazaeli M Yatsynovich Y Galectin-3 is associated with cardiac fibrosis and an increased risk of sudden death. Cells. 2023;12:1218. 10.3390/cells12091218 37174619 PMC10177039

[86] Sagris M Theofilis P Antonopoulos AS Tsioufis K Tousoulis D. Telomere length: A cardiovascular biomarker and a novel therapeutic target. Int J Mol Sci. 2022;23:16010. 10.3390/ijms232416010 36555658 PMC9781338

[87] van Duijvenboden S Nelson CP Raisi-Estabragh Z Ramirez J Orini M Wang Q Leucocyte telomere length and conduction system ageing. Heart. 2025;111:314–20. 10.1136/heartjnl-2024-324875 39689933 PMC12015050

[88] Martin-Ruiz CM Baird D Roger L Boukamp P Krunic D Cawthon R Reproducibility of telomere length assessment: an international collaborative study. Int J Epidemiol. 2015;44:1673–83. 10.1093/ije/dyu191 25239152 PMC4681105

